# Non-Adherence to Antidepressant Treatment and Related Factors in a Region of Spain: A Population-Based Registry Study

**DOI:** 10.3390/pharmaceutics14122696

**Published:** 2022-12-02

**Authors:** M. Aránzazu Pedrosa-Naudín, Eduardo Gutiérrez-Abejón, Francisco Herrera-Gómez, Diego Fernández-Lázaro, F. Javier Álvarez

**Affiliations:** 1 Pharmacy Directorate, Castilla y León Health Council, 47007 Valladolid, Spain; 2 Pharmacological Big Data Laboratory, Faculty of Medicine, University of Valladolid, 47005 Valladolid, Spain; 3 Transplantation Center, Faculty of Medicine, Lausanne University Hospital, University of Lausanne, CH-1011 Lausanne, Switzerland; 4 Department of Kidney Resuscitation and Acute Purification Therapies, Complejo Asistencial de Zamora, Sanidad de Castilla y León, 49022 Zamora, Spain; 5 Department of Cellular Biology, Histology and Pharmacology, Faculty of Health Sciences, Campus of Soria, University of Valladolid, 42003 Soria, Spain; 6 Neurobiology Research Group, Faculty of Medicine, University of Valladolid, 47005 Valladolid, Spain; 7 CEIm, Hospital Clínico Universitario de Valladolid, 47003 Valladolid, Spain

**Keywords:** antidepressants, medication adherence, drug utilization, polypharmacy, depression, anxiety, mental disorders, psychotropic drugs

## Abstract

Antidepressants are a commonly prescribed psychotropic medication, and their use has increased in recent years. Medication non-adherence in patients with mental disorders is associated with worse health outcomes. A population-based registry study to assess antidepressant non-adherence during 2021 has been carried out. An indirect method based on the medication possession ratio (MPR) has been utilized. Patients with a MPR under 80% were classified as non-adherent. A multivariate logistic regression to identify non-adherence predictors has been used, considering sociodemographic (age, sex, institutionalization and urbanicity) and health related variables (diagnostics, antidepressant class, multiple prescribers, and polypharmacy). In 2021, 10.6% of the Castile and Leon population used antidepressants. These patients were institutionalized (7.29%), living in urban areas (63.44%), polymedicated with multiple prescribers (57.07%), and using serotonin selective reuptake inhibitors (SSRIs) (54.77%), other antidepressants (46.82%) or tricyclic antidepressants (TCAs) (13.76%). Antidepressants were prescribed mainly for depression (36.73%) and anxiety (29.24%). Non-adherence to antidepressants was more frequent in men (20.56%) than in woman (19.59%) and decreased with increasing age (32% up to 17 years old vs. 13.76% over 80 years old). TCAs were associated with the highest prevalence of non-adherence (23.99%), followed by SSRIs (20.19%) and other antidepressants (18.5%). Predictors of non-adherence in patients on antidepressants were: living in urban areas, using TCAs, and pain occurrence. Non-adherence to antidepressants decreases with aging. Being female, institutionalization, being polymedicated and having depression/anxiety alongside another psychiatric diagnosis are protective factors against non-adherence. The MPR is a robust indicator for the clinician to identify non-adherent patients for monitoring, and adopt any necessary corrective actions.

## 1. Introduction

According to the Global Burden of Diseases, Injuries, and Risk Factors Study 2019 [1], mental disorders accounted for a large proportion of the burden worldwide, to which depression disorders contributed with a prevalence of 301.4 million cases, and anxiety disorders with 279.6 million affected people—the 13th and 24th leading causes of disability-adjusted life-years (DALY), respectively. Those findings will be far from improving in the near future in view of the substantial increments in the prevalence of such diseases estimated globally to have been caused by COVID-19 [2] (27.6 % increase for major depressive disorder and 25.6% for anxiety disorders for the first outbreak in 2020 alone, with the pandemic still on-going).

Antidepressants, along with benzodiazepines, are the most frequently prescribed psychotropic drugs, ranking first in the general population and second in the elderly [3,4]. Use of antidepressants has experienced a great increase over time, corresponding to an average consumption increase per year of 3.5% from 2008 to 2019 [5]. Estimations in Spain point out that 8.56% of its population used at least one antidepressant from 2015 to 2018, reporting a 21.18% raise in the consumption of such medicines over this period of time [3].

According to the European labeling, antidepressants are indicated for the treatment of not only both mood disorders mentioned before, but other mental disorders also (insomnia, eating disorders, addictions, etc.) and pain of different etiologies (chronic neuropathic pain and migraine). On the other hand, antidepressants are often used “off-label” in conjunction with antipsychotics for the treatment of schizophrenia [6]. These conditions often need long-term treatment in order to achieve successful outcomes; as in the case of major depressive disorders, treatment objectives are the improvement of symptoms, avoiding relapse and preventing recurrence [7].

Different terms have been used to define medication-taking: adherence, compliance, and concordance among others. Osterberg and Blaschke [8] defined adherence to a medication regimen as “the extent to which patients take medications as prescribed by their health care providers”. Historically, the word “adherence” has been preferred because “compliance” ignores the therapeutic alliance between patient and physician, i.e., it is based on the patient’s passive obedience to the physician’s indications. Additionally, “concordance” is defined as the relationship between reported medication taking and prescription instructions [9].

Focusing on pharmacologic treatment, mental health patients present with a variety of factors [10]—including anxiety, depression, stress, cognitive problems such as Alzheimer’s, and other psychiatric disorders affecting medication taking behaviors in a negative manner—such that low or non-adherence is a well-known issue among these patients and it indeed has been independently associated with an increased risk of health care resource utilization [11] and death [12]. In general, non-adherence rates to treatment for chronic diseases are around 50% [13], and mental illness is one of the main underlying factors for non-adherence to chronic treatments [14].

Different factors have been associated as potentially affecting non-adherence to prescribed medication. These include sociodemographic factors (age, sex, educational status, cultural context, the nature of employment, unemployment); clinical factors (side-effects, lack of insight about their illness and treatment, comorbidity, medication efficacy, medication duration, and complexity of the prescribed medication); health system related factors (lack of free access to medicine, inadequate patient–physician or –therapist relationship); substance abuse; patient attitude toward medication; patient’s self-perceived stigma; lack of social and family support; and medication cost [15,16,17,18,19,20].

Medication adherence is an important measurable indicator of the overall quality of care and is a common element for evaluating and improving healthcare delivery by policy makers [20,21,22]. Several methods to assess medication adherence have been developed and can be classified into two groups: direct methods—by direct observation or pharmacokinetic monitoring; and indirect methods—based on questionnaires, electronic devices or pharmacy records [23]. The measurement of adherence is dependent on the method used, and each approach has advantages and disadvantages as well as different effectiveness for its application to chronic diseases [24].

A recent study in Canada, which associated antidepressant prescription filling with indications, found that non-adherence to antidepressants was higher in patients suffering from depressive disorders (36.7%) and pain (39.3%) [4]. Antidepressant non-adherence is addressed by many studies conducted in different settings, but mainly when used to treat depressive disorders, with values around 50% [25,26,27], depending on the time period of measurements and the indicator used. In Spain, a previous study [28,29] revealed a non-adherence to antidepressants between 28.6% and 48.5%, regardless of the method used. However, the sample in those studies was small, 185 [29] and 370 [28] patients, respectively. Therefore, to have more exact information on non-adherence to antidepressants in Spain, a real-world study is considered necessary, reflecting the whole antidepressant-using population.

Studies to measure medication adherence are clinically relevant especially in the case of chronic diseases. Non-adherence is associated with an increased risk of health impairment, side effects and increased mortality. In addition, non-adherence carries an economic burden due to increased use of health services [30]. Finally, identifying non-adherent patients will enable the physician to support and re-engage the patient with the appropriate use of antidepressants.

According to different studies, antidepressant use [3,31] and non-adherence to antidepressants [32] is higher in women than in men. Based on this hypothesis, it would be appropriate to determine if differences between sexes exist in terms of their sociodemographic and health-related characteristics and mental health diagnoses.

The main aim of our study is to assess non-adherence prevalence among patients on antidepressants, using pharmacy records, comparing by age and sex when relevant. In addition, main predictors for non-adherence were analyzed for each antidepressant class.

## 2. Materials and Methods

### 2.1. Real-World Study Details

We carried out an epidemiological, population-based registry study. The Strengthening the Reporting of Observational Studies in Epidemiology (STROBE) [33] and the Reporting of studies Conducted using Observational Routinely-collected Data (RECORD) [34] statements were followed, in order to provide real-world evidence that adequately addresses the research topic.

This study took place in Castile and Leon, a region of Spain with a population of 2,327,420 inhabitants, and focused on those patients who used at least one antidepressant during 2021. Our data source was CONCYLIA (http://www.saludcastillayleon.es/portalmedicamento/es/indicadores-informes/concylia, accessed on 5 May 2022), the pharmacy information system for Castile and Leon, which integrates information on medicines dispensed at pharmacies and reimbursements by the National Health System in Spain. Furthermore, CONCYLIA is integrated into the Castile and Leon electronic prescription system, so it includes prescription records as well. Prescription and dispensing data are integrated through the patient identification code (PIC), which allows patient anonymization. The PIC can also be used to obtain sociodemographic and health data including diagnoses. On the other hand, data on drug use in the in-patient care setting and on private prescriptions are not available in CONCYLIA. Ultimately, the validity and reliability of the CONCYLIA database is very high as both prescription and dispensing data are integrated. In addition, the inconsistencies between these data are reviewed monthly by health inspectors.

Since a real scenario was involved, as in previous manuscripts by our group [3,35,36,37,38,39,40], all dispensations were considered equivalent to consumption. In Castile and Leon, medicines can be prescribed acutely or chronically. Most antidepressants are prescribed on a chronic basis, for 6 to 12 months.

Access to the information was permitted by the Castile and Leon Health Council Pharmacy Directorate, and the East Valladolid Area Ethics Committee approved the study protocol on the 24 February 2022 (reference number PI 22-2622).

### 2.2. Variables

To assess antidepressant adherence, we used the refill adherence measure which was quantified using the medication possession ratio (MPR), an indirect method based on pharmacy records. MPR was measured for each patient considering the days of supply during a specific monitoring period (1 year) divided by the number of days since the first dispensing until the end of the monitoring period [41]. MPR was estimated according to the prescribing and dispensing records. In addition, MPR was assessed for each antidepressant used by the patient and then for each type of antidepressant. Lastly, MPR is considered a continuous variable and is expressed as a percentage.

In this regard, we categorized patients as non-adherent when their MPR was under 80%, which is the most commonly accepted cut-off point for this parameter in the literature [42,43]. Furthermore, when appropriate, we report results by adherence level, stratified as null (<20%), poor (20–49%), moderate (50–79%) and adherent (>80%), as seen in other studies [12].

Moreover, we assessed adherence over the short, medium and long terms, setting time cut-offs of 0–2 months, 2–8 months and >8 months, respectively; matching them with successful outcomes with antidepressants when these are reached [7].

Other collected variables were sociodemographic, such as age, sex, institutionalization and urbanicity; and health related, such as diagnostics, antidepressant class, multiple prescribers, and polypharmacy.

Throughout the study, age has been considered as a continuous variable. However, it was considered interesting to observe non-adherence to antidepressants according to age group, converting the variable into a categorical variable. The age groups were divided as follows: 0–17, children and adolescents; 18–64, young and middle-aged adults; 65–79, older adults; and ≥80, elderly. The division between 17 and 18 years of age was made so as to observe if the legal age of majority, which in Spain is 18 years of age, influences non-adherence to antidepressants. Moreover, the division of ≥80 years of age has been made because the number of institutionalized patients increases after that age. For this reason, the authors considered it to be interesting to observe whether the presence of a caregiver influences non-adherence to antidepressants.

Urbanicity refers to the impact of living in urban areas at a given time [44]; for the purpose of this study, we defined those locations with 2500 or more inhabitants as urban.

Diagnostics were categorized as follows: depression, anxiety, depression and anxiety, pain of different etiologies (neuropathic pain, cephalalgia and migraine) and other psychiatric disorders. This was done in line with the International Classification of Diseases-10 (ICD-10) in order to encompass them within the approved indications of the related drugs, according to European labeling [45].

Regarding the antidepressant class, these medicines are divided into five pharmacological subgroups as per the ATC (Anatomical Therapeutic Chemical) classification system [46]. Considering that no “non-selective monoamine oxidase A inhibitors” are available in the Spanish market and data on “monoamine oxidase A inhibitors” were negligible, the three classes considered for the analyses were: “non-selective monoamine reuptake inhibitors” or tricyclic antidepressants (TCAs); “serotonin selective reuptake inhibitors” (SSRIs); and “other antidepressants” (Supplementary Table S1).

We considered patients to have multiple prescribers when their medications were prescribed by three or more different physicians [47]. Finally, for polypharmacy, we adopted the most commonly applied definition as recognized by the WHO, namely the concomitant use of five or more medications [48].

### 2.3. Statistical Analysis

Descriptive results are expressed as prevalences or percentages with an accompanying 95% confidence interval (95% CI), or as means with their standard deviation (SD). The initial assumption for the normal distribution was verified by the Kolmogorov–Smirnov and Shapiro–Wilk tests.

Differences between continuous variables were calculated using Student’s t-test (t); and for those between categorical variables, Pearson’s chi-squared test (χ²) was used.

To identify predictors of non-adherence to antidepressants, a multivariate logistic regression was performed. The results are expressed as adjusted odds ratios (AOR) with the corresponding 95% CI. Variables included in the model were those already described: sociodemographic (age, sex, institutionalization and urbanicity) and health related (antidepressants class, diagnostics, multiple prescribers, and polypharmacy). In addition, a collinearity study was performed. In light of the analysis, the depression, anxiety and depression and anxiety variables were found to be collinear. To avoid this collinearity, using the Principal Component Analysis (PCA) method, these variables have been grouped into a single predictor, depression and/or anxiety. In this model, all variables, except for age, were categorical. No missing values were detected in any variable.

All statistical analyses were performed using the Statistical Package for the Social Sciences (SPSS version 24.0., SPSS Inc., Chicago, IL, USA). The level of significance was set at *p* ≤ 0.05.

## 3. Results

### 3.1. Baseline Characteristics

In Castile and Leon, 10.6% of the population used at least one antidepressant during 2021, with a prevalence of usage more than twice as frequent in females (14.78%) than in males (6.25%). The use of these drugs increased with age reaching its maximum in those over 90 years old (y.o.), with 33.99% of this age group being prescribed an antidepressant.

Table 1 shows the characteristics, differenced by sex, of patients in treatment with antidepressants in 2021. Mean age of patients was 64.32 ± 18.66 years. Other descriptives show that less than 10% of the population was institutionalized and 63.44% lived in urban areas. Most patients in treatment with antidepressants were polymedicated (76.04%) and more than a half had multiple prescribers (57.07%).

On some occasions, a single patient was prescribed with more than one antidepressant in 2021, therefore the mean was 1.28 ± 0.59. SSRIs were the most prescribed (54.77%) followed by “other antidepressants” (46.82%), with TCAs being the less markedly prescribed (13.76%). With regards to diagnostics related to antidepressant prescription, depression was the most reported in general terms (36.73%), with anxiety close behind (29.24%). SSRIs were mainly used for depression (32.66%) and so were “other antidepressants” (32.91%), whereas pain was the principal diagnostic for TCAs (48.94%). As seen in Table 1, differences regarding sex were observed for all sociodemographic, health related characteristics and mental health diagnostics.

### 3.2. Non-Adherence to Antidepressants

Focusing on non-adherence, nearly one fifth (19.87%) of the population on antidepressants was non-adherent to these medications, this behavior being slightly more common in men than in women (20.56% vs. 19.59% respectively; *p* = 0.001). Non-adherence prevalence decreases as age increases, dropping from 32% (95% CI: 31.8–32.2) in the 0–17 years age group to 13.76% (95% CI: 13.61–13.91) in those over 80 (Supplementary Table S2). As can be observed in Table 2, superior non-adherence prevalence values were reported for patients living in urban areas who were not institutionalized, having less than three prescribers, taking less than five drugs, being on TCAs and having a diagnostic for pain.

**Figure 1:**
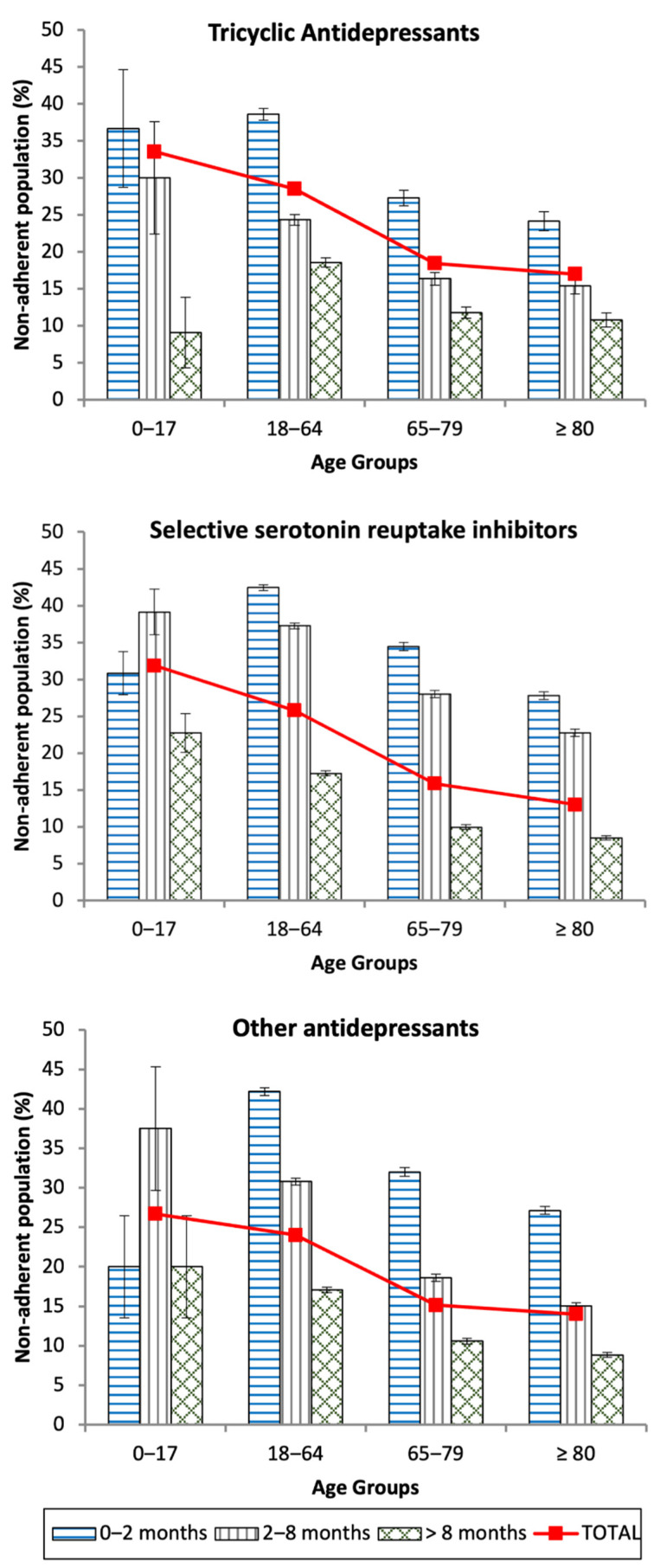
Antidepressant non-adherence prevalence among the Castile and Leon (Spain) population by length of treatment and age groups.

Figure 1 represents non-adherence prevalence distributions over age groups, for every antidepressant class, focusing on the length of treatment. As can be observed, non-adherence prevalence rates regress to lesser values as treatments increase in length. An exception to this tendency is the group from 0 to 17 y.o., as non-adherence is higher in those taking SSRIs and “other antidepressants” in the medium term.

In general, most patients categorized as non-adherent to antidepressants, around 80%, had moderate levels of adherence, followed by 17% with poor adherence and 3% being completely non-adherent. This tendency is sustained in all groups and characteristics analyzed, as it can be observed in Supplementary Table S2.

### 3.3. Risk Factor for Non-Adherence to Antidepressants

Those patients living in urban areas (AOR = 1.31, 95% CI 1.27–1.36), using TCAs (AOR = 1.48, 95% CI 1.39–1.57) and being diagnosed with pain of different etiologies (AOR = 1.3, 95% CI 1.23–1.38) are more likely to be non-adherent to treatment with antidepressants; whereas indicators that favor adherence to antidepressants are: older age (AOR = 0.94, 95% CI 0.94–0.94), being a female (AOR = 0.91, 95% CI 0.88–0.94), being institutionalized (AOR = 0.32, 95% CI 0.27–0.38), using five or more medications (AOR = 0.78, 95% CI 0.76–0.81) or having depression/anxiety (AR = 0.77, 95% CI 0.7–0.84) and another psychiatric diagnosis (AOR = 0.67; 95% CI 0.63–0.72) (Table 3).

## 4. Discussion

This study shows that antidepressants in Castile and Leon have a large prevalence of use as, approximately, one in ten people of the general population is taking at least one antidepressant, reaching one in three in those over 90 y.o.; women being the most frequent users. In 2021, one-fifth of patients on antidepressants were non-adherent to these medications, with slightly worse results in males. Non-adherence prevalence decreased as the population gets older, and also with increasing treatment length. Living in urban areas, being male, using TCAs and a pain diagnostic were factors affecting non-adherence.

The general prevalence of antidepressant use in our regional area is higher than that described in other countries in Europe [49,50], possibly because of greater difficulties in accessing healthcare facilities in other countries, leading to underdiagnosis of mental illnesses. The rates of antidepressant use in older adults and in women are consistent with the results observed in other studies [3,31]. Fundamentally, the prevalence of depression in this segment of the population is because physical dysfunction and low personal control add to personal and status losses [51].

The values of non-adherence to antidepressants observed in our study were lower than in previous studies conducted in other countries [25,26,27] and also than in other studies conducted in Spain [28,29]. These differences may be due to the different methods used for estimating non-adherence.

An observational study evaluating the impact of age on adherence to antidepressants concluded that this factor is a significant predictor of the behavior [32], as found in our model. This can be explained by the fact that the elderly are supervised to a greater extent than other adults usually are. The use of antidepressants in patients under 17 years of age is controversial and sometimes contraindicated. Nevertheless, the use of antidepressants in this age group in Castile and Leon is very low, 0.68%, and corresponds mainly to patients over 8 years of age. From that age, antidepressants such as SSRIs could be indicated, albeit always using the lowest effective dose.

In the initial hypothesis, it was assumed that both use and non-adherence to antidepressants was higher in women than in men [3,31,32]. In our study, although the prevalence of antidepressant use is double in women than in men, a slightly higher non-adherence has been observed in men than in women. In addition, being a woman was identified as a protective factor against non-adherence. However, in another study [52], the opposite was observed, with higher non-adherence in women than in men. In short, we did not identify sex as an independent factor to predict non-adherence to antidepressants, but as evidence points out, it could be a sex related phenomenon to some extent [32].

As expected, non-adherence prevalence in institutionalized patients was lower than in the general population. However, it should be noted that even more than 10% of our patients living in care homes were non-adherent to antidepressants. Furthermore, institutionalization was identified as a barrier to adherence to the class referred to as “other antidepressants”. This might be due to two main causes. One of them could be inappropriate prescriptions through electronic prescribing systems, such as failure to register the stopping date or prescribing doses for the general population instead of specific doses for the elderly [53]. The other one could be due to the fact that a minority of institutionalized patients still manage their medication on their own, and are not closely supervised on this issue [54].

In our model, urbanicity promoted non-adherence to antidepressants. Similarly, urbanicity also favored non-adherence to non-antidepressant medications in another published study [55]. However, we would expect patients living in urban areas to have better adherence outcomes to antidepressants than rural-dwelling ones, due to an easier geographic accessibility to pharmacies. A possible reason for our finding is that the workload of healthcare professionals attending rural areas in our region may have increased precisely so as to enhance communication with and better knowledge of individual patients, resulting in promoting adherence to medications more effectively. Nevertheless, other evidence has suggested that living in urban or rural locations makes no difference in adherence [56]; thus, the influence of urbanicity on medication adherence remains unclear. Due to our findings and the available literature, the urbanicity variable appears to be inadequate for measuring non-adherence to antidepressants, as it seems to be influenced by the distribution of the population in terms of gender and age more than by geographic area [56].

Our results point out polypharmacy as an independent factor preventing non-adherence to antidepressants. This is against the results reached in a recent comprehensive literature review [57], but coincident with a similar study conducted in another region in Spain associating polypharmacy with increased adherence to antidepressants [58]. However, other studies seem to indicate polypharmacy contributes to non-adherence in elderly adults [59]. An explanation for our findings may be that in polymedicated elderly patients admitted to nursing homes, medication intake is supervised by a caregiver and, thus, the rate of non-adherence is lower. On the other hand, the prevalence of polypharmacy in the elderly is increasing [60], particularly in patients with mental disorders [61]. This factor is of high importance since the increase in prescribed medications and the regimen complexity is associated with lower medication adherence [62]. In addition, polypharmacy has been associated with treatment complications, increased risk of side effects and increased costs [61].

To our knowledge, there is no specific research evaluating the influence of multiple prescribers on non-adherence to antidepressant treatment in the general population. A small study conducted in older community-dwelling patients supports the hypothesis that an increase in the number of prescribing physicians has an impact on self-reported adverse drug reactions which, in turn, has been described as an independent factor contributing to non-adherence [47]. Moreover, Hansen et al. [63] conclude that having less prescribers may improve medication adherence in complex patients with cardiometabolic conditions, such as hypertension and dyslipidemia.

In general terms, the prevalence of non-adherence to antidepressants decreases as the duration of treatment increases. Indeed, this fact is not surprising, since one of the main causes of non-adherence is side effects [20,64], which are more frequent at the initiation of treatment, especially in the case of TCAs [65].

In our study, “other antidepressants” had slightly better outcomes for adherence than SSRIs, although it remains within the same magnitude. A study assessing adherence and persistence to antidepressants, focused on patients with a major depressive disorder, stated that those on serotonin and norepinephrine reuptake inhibitors (SNRI), included in “other antidepressants” class, were more likely than those on SSRIs to carry on taking the drug over the period of a year [66]; whereas a review of the literature points out that prevalence of non-adherence related to antidepressants other than SSRIs is higher, because of the benefit–risk ratio associated to this class [57].

The class with the worse values in our report was TCAs. With respect to this, a retrospective analysis assessing adherence to TCAs, through urine testing, revealed that 66% of the patients were adherent to these drugs when used for treating neuropathic pain [65]. In our study, TCAs were mostly used for pain, although the outcome for adherence was better. The difference in results is attributable to using different methods of measuring adherence and indicates that ours may underestimate non-adherence rates. On the other hand, TCAs have long half-lives, up to 90 h in some cases, leading to the question whether the 80% MPR cut-off point has any clinical transcendence in pain, or whether lower values could be considered to be more appopriate [4].

As discussed above, antidepressants are prescribed for several diagnoses. Not surprisingly, our findings are in accordance with other available reports, with depressive disorders being the primary indication for prescribing SSRIs and “other antidepressants”, and pain in the case of TCAs [4].

Prevalence of non-adherence for each diagnosis is in consonance with the antidepressant used: it is thus higher for pain—since TCAs are medicines that are frequently associated with adverse effects and intolerance; and lower in depressive disorders and anxiety—since SSRIs and “other antidepressants”, mainly SNRIs, are usually safer and better tolerated, particularly in the elderly [67]. Furthermore, many antidepressants are being discontinued because of serious adverse events, which may contribute non-adherence [68].

The proportion of patients in Castile and Leon not using their antidepressants properly is lower than that observed in other localities, but still quite significant. Non-adherence to these drugs contributes to relapse and may lead to dose titration or treatment augmentation with other agents without an actual clinical need [52].

Finally, the main limitations to our study must be mentioned. Factors that may explain the non-adherence that have not been considered because they are not available, include intrapersonal (empowerment, attitude towards medication, insight, self-stigma, working status, etc.), interpersonal (bond of trust, communication skills of the prescriber, attitudes towards mental health of the prescriber, specialty of the prescriber, use of shared decision making, caregivers’ influence, etc.) and contextual (type of center, etc.). In addition, there are no data available on other neurological and psychiatric comorbidities that could influence adherence to antidepressants. Another limitation of the study is that an indirect method, using pharmacy records, was used to measure non-adherence to antidepressants assuming that filling is actually equivalent to medication taking, so non-adherence prevalence may have been underestimated [23]. Furthermore, we lack the data on antidepressant use in hospital settings and on private prescriptions, as this information is not available in our data source, CONCYLIA. However, as the sample size is large and the antidepressants covered are prescription-only medicines, the biases occurring using dispensing data are not considered to be relevant. As seen in many studies, the 80% cut-off point for the MPR is widely accepted for considering patient behavior as adherent, yet it is not a universal score [42], so values below could be acceptable in some circumstances [4]. Lastly, in contrast to other publications [57], our findings show polypharmacy as a protective factor against non-adherence to SSRIs and “other antidepressants”, which may seem inconsistent. However, in another Spanish study [58], polypharmacy was also identified as a protective factor for non-adherence.

## 5. Conclusions

One in ten inhabitants of Castile and Leon, the largest region in Spain, used an antidepressant in 2021, with a prevalence of non-adherence lower to that reported in other settings, but still a relevant healthcare issue to consider.

Non-adherence prevalence decreases with ageing, so having an elderly population as in our study could magnify adherence values to antidepressants in general terms, as found in our results. It has been observed that other factors such as living in rural locations, being female, institutionalization, being polymedicated and having depression/anxiety and another psychiatric diagnosis are protecting factors against non-adherence to antidepressants. The worst outcomes for adherence were detected for TCAs, but this finding might not be relevant for mental health diseases since TCAs were mainly used for pain, an approved indication as per the European labeling. In our study, no differences between adherence to SSRIs and to “other antidepressants” have been found in patients with depressive disorders.

MPR is an appropriate indicator to aid health care systems establish general strategies to enhance adherence to antidepressants. Because it is an indirect method, this measure could underestimate non-adherence, so it is important that healthcare professionals assess adherence behavior on an individual basis. In this regard, several reports identifying non-adherent patients are generated by CONCYLIA for submission to their general practitioner. With these reports, the physician can verify if the patient is really non-adherent to antidepressants. In this case, if considered necessary by the physician, the patient is scheduled for a medical consultation to adopt the necessary corrective actions.

Finally, since this is a population-based registry study, it is not possible to use a direct method to measure medication adherence. Notwithstanding this, the method used in our study provides a sufficiently robust indicator to identify patients non-adherent to antidepressants. In the future, to obtain more conclusive results, a replication of the study with a smaller sample size and using different methods would be desirable.

## Figures and Tables

**Table 1:** Baseline characteristics of the Castile and Leon (Spain) population on antidepressants.

	Total	Male	Female
**N**	246,718	71,340	175,378
**Sociodemographic characteristics**			
*Age (mean ± SD)*	64.32±18.66	62.68 ± 18.71	64.99 ± 18.60
*Distribution by age groups (%)*			
0–17	0.68	0.74	0.65
18–64	48.32	51.96	46.84
65–79	25.82	25.01	26.15
≥80	25.19	22.3	26.36
*Institutionalized % (95% CI)*	7.29	7.63	7.16
*Urbanicity % (95% CI)*	63.44	61.67	64.16
**Health related characteristics (%)**			
*Polypharmacy*	76.04	71.84	77.74
*Multiple prescribers*	57.07	55.99	57.5
*Antidepressant class*			
SSRIs	54.77	51.32	56.18
Other antidepressants	46.82	51.23	45.02
TCAs	13.76	12.14	14.42
**Mental health diagnostics (%)**			
*Depression*	36.73	37.03	36.62
*Anxiety*	29.24	30.55	28.7
*Depression and Anxiety*	22.06	18.17	23.65
*Pain (different etiologies)*	10.04	8.04	10.86
*Other psychiatric diagnosis*	7.94	11.33	6.57

Abbreviations: SD, standard deviation; CI, confidence interval; SSRIs, Selective Serotonin Reuptake Inhibitors; TCAs, Tricyclic Antidepressants.

**Table 2:** Antidepressant non-adherence prevalence (%) among the Castile and Leon (Spain) population.

		Non-Adherence Prevalence % (95% CI)	*p*
**Sociodemographic Characteristics**			
*Sex*			
	Male	20.56 (20.39–20.74)	0.001
	Female	19.59 (19.42–19.76)	
*Urbanicity*			
	Yes	20.92 (20.75–21.1)	0.001
	No	17.96 (17.79–18.12)	
*Institutionalized*			
	Yes	13.61 (13.47–13.76)	0.001
	No	20.39 (20.22–20.57)	
**Health related characteristics**			
*Multiple prescribers*			
	Yes (≥3 prescribers)	19.19 (19.02–19.36)	0.001
	No (<3 prescribers)	20.8 (20.63–20.97)	
*Polypharmacy*			
	Yes (≥5 drugs)	18.43 (18.27–18.6)	0.001
	No (<5 drugs)	24.85 (24.66–25.03)	
*Antidepressant class*			
	TCAs	23.99 (23.8–24.17)	0.001
	SSRIs	20.19 (20.01–20.36)	
	Other antidepressants	18.5 (18.34–18.67)	
**Mental health diagnostics**			
*Pain (different etiologies)*		24.81 (24.62–24.99)	0.001
*Anxiety*		21.55 (21.37–21.72)	
*Depression and Anxiety*		19.47 (19.3–19.64)	
*Depression*		17.83 (17.67–17.99)	
*Other psychiatric diagnosis*		17.75 (17.59–17.92)	

Abbreviations: 95% CI: confidence interval, SSRIs, Selective Serotonin Reuptake Inhibitors, TCAs: Tricyclic Antidepressants.

**Table 3:** Risk Factors for non-adherence to antidepressants among the Castile and Leon (Spain) population during 2021.

	AOR (95% CI)	*p*
**Sociodemographic characteristics**		
*Age*	0.94 (0.94–0.94)	0.001
*Gender (Female)*	0.91 (0.88–0.94)	0.001
*Urbanicity*	1.31 (1.27–1.36)	0.001
*Institutionalized*	0.32 (0.27–0.38)	0.001
**Health related characteristics**		
*Polypharmacy (≥5)*	0.78 (0.76–0.81)	0.001
*Multiple prescribers (≥3)*	0.97 (0.93–1.01)	0.322
*Antidepressants class*		
SSRIs	1 (0.96–1.04)	0.99
Other antidepressants	0.95 (0.91–1)	0.23
TCAs	1.48 (1.39–1.57)	0.001
**Mental health diagnostics**		
*Depression and/or anxiety*	0.77 (0.7–0.84)	0.001
*Pain (different etiologies)*	1.3 (1.23–1.38)	0.001
*Other psychiatric diagnosis*	0.67 (0.63–0.72)	0.001

Abbreviations: SD, standard deviation; CI, confidence interval; SSRIs, Selective Serotonin Reuptake Inhibitors; TCAs, Tricyclic Antidepressants.

## Data Availability

Restrictions apply to the availability of these data. Data were obtained from regional health authorities (Gerencia Regional de Salud (GRS)) and may be requested from conciertofco@saludcastillayleon.es (GRS).

## Supplementary Materials

The following supporting information can be downloaded at: https://www.mdpi.com/article/10.3390/pharmaceutics14122696/s1, Table S1: Antidepressants available in Castile and Leon; Table S2: Population characteristics distribution across adherence levels to antidepressants in Castile and Leon.
