# Supplementary material for: Non-Adherence to Antidepressant Treatment and Related Factors in a Region of Spain: A Population-Based Registry Study
Source: Pharmaceutics. 2022 Dec 2;14(12):2696. doi: 10.3390/pharmaceutics14122696 (PMC9782667; doi:10.3390/pharmaceutics14122696)
Supplement: Supplementary file 1 [file pharmaceutics-14-02696-s001.zip › Supplementary_Material.pdf]

Supplementary Table S1. Antidepressants available in Castile and Leon.

Antidepressant class	ATC Code	Drug
Non-selective monoamine reuptake inhibitors	N06AA02	Imipramine
	N06AA04	Clomipramine
	N06AA06	Trimipramine
	N06AA09	Amitryptiline
	N06AA10	Nortiptyline
	N06AA12	Doxepine
	N06AA21	Maprotiline
Selective serotonin reuptake inhibitors	N06AB03	Fluoxetine
	N06AB04	Citalopram
	N06AB05	Paroxetine
	N06AB06	Sertraline
	N06AB08	Fluvoxamine
	N06AB10	Escitalopram
Monoamine oxidase A inhibitors	N06AG02	Moclobemide
Other antidepressants	N06AX03	Mianserin
	N06AX05	Trazodone
	N06AX11	Mirtazapine
	N06AX12	Bupropion
	N06AX14	Tianeptine
	N06AX16	Venlafaxine
	N06AX18	Reboxetine
	N06AX21	Duloxetine
	N06AX22	Agomelatine
	N06AX23	Desvenlafaxine
	N06AX26	Vortioxetine

Abbreviations: ATC; Anatomical, Therapeutical and Chemical Classification.

Supplementary Table S2. Population characteristics distribution across adherence levels to antidepressants in Castile and Leon.

	Non-adherent			Adherent
**Adherence level**	**None (<20)**	**Poor (20 - 49)**	**Moderate (50 - 79)**	**(≥ 80)**
**Total**	0.39 (0.36-0.41)	3.47 (3.39-3.55)	16.01 (15.86-16.17)	80.13 (79.96-80.3)
**Sociodemographic characteristics**				
Sex				
Male	0.44 (0.41-0.47)	3.66 (3.58-3.74)	16.47 (16.31-16.63)	79.44 (79.26-79.61)
Female	0.36 (0.34-0.39)	3.39 (3.32-3.47)	15.83 (15.68-15.99)	80.41 (80.24-80.58)
Age group				
0 - 17	0.62 (0.59-0.65)	5.32 (5.22-5.42)	26.06 (25.88-26.25)	68 (67.8-68.2)
18 - 64	0.55 (0.51-0.58)	4.72 (4.63-4.81)	20.26 (20.09-20.44)	74.47 (74.28-74.66)
65 - 79	0.26 (0.24-0.28)	2.61 (2.54-2.67)	12.93 (12.79-13.07)	84.21 (84.05-84.36)
≥ 80	0.23 (0.21-0.25)	2.1 (2.04-2.16)	11.43 (11.3-11.57)	86.24 (86.09-86.39)
Institutionalized	0.15 (0.13-0.16)	1.87 (1.81-1.92)	11.6 (11.46-11.74)	86.39 (86.24-86.53)
Urbanicity	0.4 (0.38-0.43)	3.71 (3.63-3.79)	16.81 (16.65-16.97)	79.08 (78.9-79.25)
**Health related characteristics (95% CI)**				
Polypharmacy (≥ 5 drugs)	0.37 (0.35-0.4)	3.14 (3.06-3.21)	14.92 (14.77-15.07)	81.57 (81.4-81.73)
Multiple prescribers (≥ 3)	0.35 (0.32-0.38)	3.21 (3.12-3.31)	15.62 (15.43-15.82)	80.81 (80.6-81.02)
Antidepressant class				
TCAs	0.6 (0.57-0.63)	4.88 (4.79-4.97)	18.51 (18.34-18.67)	76.01 (75.83-76.2)
SSRIs	0.32 (0.3-0.34)	3.31 (3.23-3.38)	16.56 (16.4-16.72)	79.81 (79.64-79.99)
Other antidepressants	0.35 (0.32-0.37)	2.84 (2.77-2.91)	15.32 (15.17-15.47)	81.5 (81.33-81.66)
**Mental health diagnostics (95% CI)**				
Pain (different etiologies)	0.69 (0.65-0.72)	5.39 (5.3-5.49)	18.73 (18.56-18.89)	75.19 (75.01-75.38)
Anxiety	0.38 (0.36-0.41)	3.76 (3.68-3.84)	17.4 (17.24-17.57)	78.45 (78.28-78.63)
Depression and Anxiety	0.34 (0.32-0.37)	2.99 (2.92-3.07)	16.13 (15.98-16.29)	80.53 (80.36-80.7)
Depression	0.31 (0.29-0.33)	2.98 (2.9-3.05)	14.54 (14.39-14.69)	82.17 (82.01-82.33)
Other psychiatric diagnoses	0.34 (0.31-0.36)	2.66 (2.59-2.73)	14.75 (14.6-14.9)	82.25 (82.08-82.41)

Abbreviations: 95% CI, confidence interval, SSRIs, Selective Serotonin Reuptake Inhibitors; TCAs: Tricyclic Antidepressants.
